# Recurrent fever and association with severe organ involvement, mortality and treatment outcomes in VEXAS syndrome: data from the AIDA Network

**DOI:** 10.3389/fimmu.2026.1753412

**Published:** 2026-03-13

**Authors:** Valeria Caggiano, Jessica Sbalchiero, Micol Frassi, Francesca Crisafulli, Ilaria Cavazzana, Andrea Hinojosa-Azaola, Eduardo Martín-Nares, Guillermo Arturo Guaracha-Basañez, Jiram Torres-Ruiz, Ewa Wiesik-Szewczyk, Anna Nowakowska-Plaza, Paolo Sfriso, Sara Bindoli, Samuele Rizzo, José Hernández-Rodríguez, Verónica Gómez-Caverzaschi, Olga Araújo, Henrique A. Mayrink Giardini, Andrés González-García, Giuseppe Lopalco, Ombretta Viapiana, Riccardo Bixio, Abdurrahman Tufan, Pravin Hissaria, Mark Beecher, Alicia Callisto, Amato De Paulis, Lorenzo Dagna, Corrado Campochiaro, Alessandro Tomelleri, Serena Bugatti, Alessandra Milanesi, Guillermo Ruiz-Irastorza, Adriana Soto-Peleteiro, Matteo Piga, Alberto Cauli, Fabrizio Conti, Chiara Cardamone, Paola Triggianese, Carmelo Gurnari, Rosetta Vitetta, Marcella Prete, Vito Racanelli, Rosaria Talarico, Francesco Ferro, Federica di Cianni, Maria Chiara Cuccaro, Adrián Mayo-Juanatey, Alessandra Renieri, Vincenzo Sorrentino, Mehmet Ali Ergun, Alberto Balistreri, Bruno Frediani, Monica Bocchia, Claudia Fabiani, Luca Cantarini, Antonio Vitale

**Affiliations:** 1 Department of Medical Sciences, Surgery and Neurosciences, Research Center of Systemic Autoinflammatory Diseases and Behçet’s Disease Clinic, University of Siena, Siena, Italy; 2 Azienda Ospedaliero-Universitaria Senese [European Reference Network (ERN) for Rare Immunodeficiency, Autoinflammatory and Autoimmune Diseases (RITA) Center], Siena, Italy; 3 Rheumatology and Clinical Immunology, Spedali Civili and Department of Clinical and Experimental Sciences, University of Brescia, [European Reference Network (ERN) for Rare Immunodeficiency, Autoinflammatory and Autoimmune Diseases (RITA) Center], Brescia, Italy; 4 Department of Immunology and Rheumatology, Instituto Nacional de Ciencias Médicas y Nutrición Salvador Zubirán, Mexico City, Mexico; 5 Department of Internal Medicine, Pneumonology, Allergology, Clinical Immunology and Rare Diseases, Military Institute of Medicine, National Research Institute, Warsaw, Poland; 6 Rheumatology Unit, Department of Medicine, University of Padua, [European Reference Network (ERN) for Rare Immunodeficiency, Autoinflammatory and Autoimmune Diseases (RITA) Center], Padua, Italy; 7 Clinical Unit of Autoinflammatory Diseases, Department of Autoimmune Diseases, Institut d’Investigacions Biomèdiques August Pi I Sunyer (IDIBAPS), Hospital Clínic of Barcelona [European Reference Network (ERN) for Rare Immunodeficiency, Autoinflammatory and Autoimmune Diseases (RITA) Center], University of Barcelona, Barcelona, Spain; 8 Rheumatology Division, Faculdade de Medicina, Hospital das Clínicas, Universidade de São Paulo, São Paulo, Brazil; 9 Systemic Autoimmune Diseases Unit, Department of Internal Medicine, Hospital Universitario Ramón y Cajal, IRYCIS, Madrid, Spain; 10 Department of Precision and Regenerative Medicine and Ionian Area (DiMePRe-J) Policlinic Hospital, University of Bari, Bari, Italy; 11 Rheumatology Unit, Department of Medicine, University and Azienda Ospedaliera Universitaria Integrata of Verona, Verona, Italy; 12 Rheumatology Unit, IRCCS Sacro Cuore Don Calabria Hospital, Negrar di Valpolicella, Verona, Italy; 13 Department of Internal Medicine, Division of Rheumatology, Gazi University Hospital, Ankara, Türkiye; 14 Department of Clinical Immunology and Allergy, Royal Adelaide Hospital, Adelaide, SA, Australia; 15 Department of Immunopathology, SA Pathology, Adelaide, SA, Australia; 16 Department of Translational Medical Sciences, Section of Clinical Immunology, University of Naples Federico II, Naples, Italy; 17 Center for Basic and Clinical Immunology Research (CISI), World Allergy Organization (WAO) Center of Excellence, University of Naples Federico II, Naples, Italy; 18 Division of Immunology, Transplants and Infectious Diseases, Università Vita-Salute San Raffaele, Milan, Italy; 19 Unit of Immunology, Rheumatology, Allergy and Rare Diseases, IRCCS Ospedale San Raffaele, [European Reference Network (ERN) for Rare Immunodeficiency, Autoinflammatory and Autoimmune Diseases (RITA) Center], Milan, Italy; 20 Department of Internal Medicine and Therapeutics, Università di Pavia, Pavia, Italy; 21 Division of Rheumatology, Fondazione IRCCS Policlinico San Matteo, [European Reference Network (ERN) for Rare Immunodeficiency, Autoinflammatory and Autoimmune Diseases (RITA) Center], Pavia, Italy; 22 Faculty of Medicine and Nursery, University of the Basque Country, Euskal Herriko Unibertsitatea (UPV/EHU), Leioa, Biscay, Spain; 23 Autoimmune Diseases Unit, Biocruces Bizkaia Health Research Institute, Barakaldo, Biscay, Spain; 24 Rheumatology Unit, Department of Medical Sciences and Public Health, University and Azienda Ospedaliero Universitaria (AOU) of Cagliari, Cagliari, Italy; 25 Department of Internal Medicine and Medical Specialties, Rheumatology Unit, Azienda Ospedaliero Universitaria (AOU) Policlinico Umberto I, Sapienza University of Rome, Rome, Italy; 26 Unità Operativa Complessa (UOC) of Internal Medicine - Rheumatology Outpatients Unit, Azienda Ospedaliero-Universitaria San Giovanni di Dio e Ruggi D’Aragona, Via San Leonardo 1, Salerno, Italy; 27 Unità Operativa Complessa (UOC) Medicina Interna - UOSD Geriatria, Università di Roma Tor Vergata, Rome, Italy; 28 Department of Biomedicine and Prevention, University of Rome Tor Vergata, Rome, Italy; 29 Department of Translational Hematology and Oncology Research, Taussig Cancer Institute, Cleveland Clinic, Cleveland, OH, United States; 30 Unit of Rheumatology, Azienda Sanitaria Locale di Vercelli (ASL VC) Sant’ Andrea Hospital, Vercelli, Italy; 31 Rheumatic and Systemic Autoimmune Diseases Unit, Department of Interdisciplinary Medicine (DIM), University of Bari Medical School, Bari, Italy; 32 Centre for Medical Sciences, University of Trento and Internal Medicine Division, Santa Chiara Hospital, Provincial Health Care Agency (APSS), Trento, Italy; 33 Rheumatology Unit, Department of Clinical and Experimental Medicine, University of Pisa, Pisa, Italy; 34 Hematology, Azienda Ospedaliera Universitaria Senese, University of Siena, Siena, Italy; 35 Department of Rheumatology, Doctor Peset University Hospital, Valencia, Spain; 36 Medical Genetics, Department of Medical Biotechnologies, University of Siena, Siena, Italy; 37 Department of Medical Biotechnologies, Med Biotech Hub and Competence Center, University of Siena, Siena, Italy; 38 Genetica Medica, Azienda Ospedaliero-Universitaria Senese, Siena, Italy; 39 Molecular Medicine Section, Department of Molecular and Developmental Medicine, University of Siena, Siena, Italy; 40 Department of Medical Genetics, Faculty of Medicine, Gazi University, Ankara, Türkiye; 41 Bioengineering and Biomedical Data Science Lab, Department of Medical Biotechnologies, University of Siena, Siena, Italy; 42 Ophthalmology Unit, Department of Medicine, Surgery and Neurosciences, University of Siena, Siena, Italy

**Keywords:** anakinra, autoinflammatory disease, JAK, personalized medicine, prognosis, tocilizumab (TCZ)

## Abstract

**Introduction:**

Recurrent febrile episodes account for one of the most frequent symptoms observed in Vacuoles, E1 enzyme, X-linked, Autoinflammatory, Somatic (VEXAS) syndrome and a key target for therapeutic intervention. Therefore, this study aims at investigating the association between recurrent febrile episodes and specific clinical manifestations, mortality and response to treatment.

**Methods:**

Data were obtained from the international AutoInflammatory Disease Alliance (AIDA) Network registry and analyzed using a Bayesian statistical approach. Posterior probabilities [P(β)] were calculated to assess the likelihood that fever was associated with clinical, laboratory, genetic, and therapeutic features.

**Results:**

In total, 87 VEXAS patients were enrolled, 65 (74.7%) of whom suffered from recurrent fever episodes. Fever episodes showed a significant association with patients’ mortality [P(β): 99.41%], as well as with major inflammatory organ involvement, including cardiac [P(β): 99.99%], lung [P(β): 99.98%], and gastrointestinal [P(β): 97.5%] involvement. The occurrence of recurrent fever episodes was associated with a negligible probability of both complete response and treatment failure [P(β) <2.5%], instead favoring a partial response [P(β) >97.5%] to conventional disease modifying anti-rheumatic drugs, Janus Kinases inhibitors, and tocilizumab. For temperatures exceeding 40 °C, using anti-interleukin-1 agents was associated with a high probability of treatment failure [P(β): 99.3%].

**Conclusions:**

febrile episodes are associated with more severe pattern of organ involvement and, accordingly, to death. Furthermore, febrile episodes could correlate with differential therapeutic responsiveness, thereby potentially serving as a valuable marker to guide the treatment strategies in VEXAS patients.

## Introduction

Vacuoles, E1 enzyme, X-linked, Autoinflammatory, Somatic (VEXAS) syndrome is a recently identified, severe autoinflammatory disorder that primarily, but not exclusively, affects adult men. It is caused by somatic mutations in the X-linked *UBA1* gene, encoding the E1 ubiquitin-activating enzyme and leading to a combination of systemic inflammation and hematologic abnormalities, often mimicking or inducing rheumatologic and hematologic diseases (1, 2). Vacuoles in bone marrow precursor cells characterize the disease and are often associated with myelodysplastic syndrome (MDS) and other hematologic disorders (3, 4).

Patients typically present in late adulthood with recurrent fever episodes, skin lesions, chondritis, arthritis, pulmonary involvement, vasculitis, and macrocytic anemia (5). Thrombosis is common, affecting up to 40% of patients (5, 6). Diagnosis relies on clinical suspicion, presence of vacuoles in bone marrow findings, and confirmation of *UBA1* mutations through genetic testing (2). The clinical spectrum is broad, and genotype-phenotype correlations are emerging, with certain mutations linked to specific organ involvement (7, 8).

Regarding treatment, glucocorticoids remain the mainstay of acute management; however, the high doses required make them unsuitable for long-term monotherapy. Conventional disease modifying anti-rheumatic drugs (cDMARDs) yield partial responses in some cases, but complete responses are rare (9). Among the biotechnological agents (bDMARDs), interleukin(IL)-1 inhibitors anakinra and canakinumab and the anti-IL-6 agent tocilizumab have proved to be at least a partial efficacy in most of cases (10, 11). Although carrying a higher risk for infections (12), the Janus Kinases (JAK) inhibitors provide partial or complete responses in most of patients (10, 13). A very promising role has been shown by the hypomethylating drug azacitidine (14), while allogeneic hematopoietic stem cell transplantation represents the only curative option, with pooled overall survival of 86%, but significant risks of graft-versus-host disease and non-relapse mortality (15).

In this context, the occurrence of recurrent febrile episodes, reported in approximately two-thirds of cases and in up to 82% of patients in smaller cohorts, accounts for one of the most frequent symptoms observed in VEXAS syndrome and a key target for therapeutic intervention (5, 16, 17). On this basis, the present study was designed to address the scientific question about the possible association between recurrent febrile episodes and specific clinical manifestations, particularly major organ inflammatory involvement affecting the lungs, gastrointestinal tract, vascular and heart, as well as their impact on mortality and the potential therapeutic implications linked to the presence and severity of such episodes.

## Methods

Demographic, genetic and clinical data were obtained from the International AutoInflammatory Disease Alliance (AIDA) Network registry dedicated to VEXAS syndrome (18). Actually, the AIDA project envisages the development of physician-driven, non-population-based international registries designed to collect real-world data on patients with rare autoinflammatory diseases. Data are entered by participating clinicians using a standardized electronic data capture system based on the Research Electronic Data Capture (REDCap) platform (19). The registry supports both retrospective and prospective observational clinical research projects. Additional details are available on the official AIDA Network website (https://aidanetwork.org/en/). All participants harbored a pathogenic or likely pathogenic mutation in the *UBA1* gene.

Patients were classified according to recurrent febrile episodes (at least one episode per year) not attributable to known factors capable of inducing an increase in body temperature, including infections, neoplastic or concomitant autoimmune diseases. Patients with febrile episodes were further stratified into three groups based on the typical body temperature reached during flares: 37.1-37.7 °C, representing patients with recurrent low-grade temperature elevations associated with clear inflammatory activity; 37.8-40 °C; and >40 °C, representing patients with marked temperature increases.

Mortality, clinical manifestations, laboratory features, specific genetic mutations, and response to therapies administered over time (cDMARDs, bDMARDs, and JAK inhibitors) were evaluated for associations with recurrent febrile episodes. Regarding mutations, the classical canonical variants (p.Met41Leu; p.Met41Val, and p.Met41Thr) were assessed individually, while all other mutations were grouped together. For treatment response, cDMARDs were evaluated as a whole (colchicine, azathioprine, cyclosporine, methotrexate altogether), with methotrexate assessed separately, as it was the only agent with a sufficiently large sample size to allow individual analysis. Similarly, among anti-IL-1 biologic agents, only the receptor antagonist anakinra was evaluated, and among JAK inhibitors, only ruxolitinib was assessed individually. JAK inhibitors were analyzed collectively (baricitinib, filgotinib, ruxolitinib, tofacitinib, upadacitinib) and separately according to their selectivity for JAK2: JAK2-selective agents (baricitinib and ruxolitinib) on one hand, and non-JAK2-selective agents (filgotinib, tofacitinib, upadacitinib) on the other. The assessment of IL-6 inhibitors corresponded to the evaluation of response to tocilizumab. Response criteria to treatments were adapted from previous AIDA studies, in which complete response, partial response, and treatment failure to cDMARDs, bDMARDs, and JAK inhibitors were defined (9, 11). In particular, complete response was defined as the resolution of VEXAS-related clinical manifestations, accompanied by normalization or only a slight increase (no more than 10% above the upper limit of the cut-off) of inflammatory parameters and no increase in the daily prednisone dosage. Partial response was defined as the persistence of clinical and laboratory manifestations with significantly reduced severity and/or frequency of acute exacerbations, as reported by patients and observed by physicians. Failure was defined as the persistence of clinical manifestations and/or insufficient reduction in inflammatory markers to meet the previous definitions.

Statistical analysis included descriptive methods such as mean, median, standard deviation (SD), interquartile range, frequency counts, and corresponding percentages. A Bayesian logistic regression framework was employed to assess the association between the presence of fever (independent variable) and each demographic, clinical, and laboratory variable, as well as specific *UBA1* mutations and the response to the different treatment approaches (each considered individually as the dependent variable). Models were fitted using the *brms* package in R Studio, which interfaces with Stan for Bayesian inference via Hamiltonian Monte Carlo. Each model estimated the posterior distribution of the log-odds of the dependent variable as a function of fever. A weakly informative normal prior (mean = 0, SD = 5) was applied to all regression coefficients to regularize estimation while avoiding strong assumptions. Each model was run using four Markov chains, each with 2,000 iterations (1,000 warm-up and 1,000 sampling steps), ensuring proper convergence and adequate exploration of the posterior distribution. The posterior probability [P(β)] of a positive (or negative) association was computed directly from the posterior draws, allowing for intuitive probabilistic interpretation of effect estimates. Odds ratios (ORs) and corresponding 95% credible intervals (CrIs) were obtained by exponentiating the posterior distributions of the regression coefficients. Associations were considered statistically significant when the 95% CrI excluded the null value (OR = 1) and/or when the P(β) exceeded 97.5% or was below 2.5%. Variables associated with fever were subsequently analyzed in subgroups stratified by body temperature during fever flares (37.1-37.7 °C, 37.8-40 °C, >40 °C) to determine whether the associations held true for fever of varying intensity.

## Results

In total, 87 VEXAS patients were enrolled. Recurrent fever episodes were reported in 65 (74.7%) of cases. The temperature during fever episodes ranged between 37 °C and 37.7 °C in 17 (19.5%) cases, while 31 (35.6%) patients had a temperature ranging between 37.8 °C and 40 °C, and 5 (5.7%) patients had a temperature exceeding 40 °C. In 12 (13.8%) cases, the maximum temperature reached during the febrile episodes was reported as unknown. The median follow-up duration was 60 (IQR: 46) months among patients with fever attacks and 56.5 (IQR: 62) months among patients with no fever attacks (p=0.59). Demographic and clinical features are summarized in **Table 1** according to the presence or absence of recurrent fever episodes.

**Table 1:** Demographic, clinical, laboratory, and genetic features of patients stratified by the presence of recurrent fever episodes.

Variables	Patients with fever, N=65	Patients without fever, N=22
Sex N	1 female and 64 males	3 females and 19 males
Age at disease onset (years), mean±SD	64.6±10.9	71.8±10.7
Age at diagnosis (years), mean±SD	68.6±10.2	76.0± 9.97
Age at enrollement (years), mean±SD	70.6±7.30	78.1±7.47
Follow-up duration al last visit (months), median (IQR)	60 (46)	56.5 (62)
Relapsing-remitting disease course, N (%)	32 (49.2)	6 (27.3)
Number of fever attacks/year, median (IQR)	4.5 (4.25)	NA
Mean duration of fever attacks (days), median (IQR)	7.5 (9.5)	NA
Concomitant haematological comorbidities, N (%)	40 (61.5)	14 (63.6)
Myelodysplastic syndrome, N (%)	31 (47.7)	13 (59.1)
Systemic comorbidities, N (%)	38 (58.5)	14 (63.6)
Deceased, N (%)	10 (15.4)	1 (4.5)
**Skin rash**, N (%)	60 (92.3)	17 (77.3)
Neutrophilic dermatosis, N (%)	18 (27.7)	3 (13.6)
Macular lesions, N (%)	8 (12.3)	3 (13.6)
Urticarial papules, N (%)	6 (9.2)	2 (9.1)
Urticarial like eruptions, N (%)	10 (15.4)	1 (4.5)
Erythema , N (%)	12 (18.5)	1 (4.5)
Eyelids edema, N (%)	6 (9.2)	0 (0)
Other, N (%)	39 (60)	10 (45.5)
**Ocular involvement**, N (%)	31 (47.7)	9 (40.9)
Uveitis, N (%)	5 (7.7)	0 (0)
Scleritis, N (%)	8 (12.3)	3 (13.7)
Episcleritis, N (%)	7 (10.8)	1 (4.5)
Periorbital edema, N (%)	11 (16.9)	3 (13.6)
**Arthritis** , N (%)	27 (41.5)	8 (36.4)
**Chondritis**, N (%)	20 (30.8)	6 (27.3)
**Gastrointestinal involvement**, N (%)	12 (18.5)	1 (4.5)
**Abdominal pain**, N (%)	11 (16.9)	1 (4.5)
**Splenomegaly**, N (%)	18 (27.7)	5 (22.7)
**Neurological involvement**, N (%)	13 (20)	3 (13.7)
**Vascular involvement**, N (%)	34 (52.3)	9 (40.9)
**Cardiac involvement**, N (%)	16 (24.6)	0 (0)
**Lymphadenopathy** , N (%)	21 (32.3)	1 (4.5)
**Thoracic pain**, N (%)	14 (21.5)	2 (9.1)
**Pleural effusion**, N (%)	10 (15.4)	1 (4.5)
**Pericardial effusion**, N (%)	4 (6.2)	0 (0)
**Lung infiltrates**, N (%)	33 (50.8)	3 (13.7)
**Kidney involvement**, N (%)	6 (9.2)	2 (9.1)
Laboratory items		
Proteinuria, N (%)	4 (6.2)	0 (0)
Anemia (all types), N (%)	60 (92.3)	21 (95.5)
Macrocytic anemia	39 (60)	15 (68.2)
Macrocytosis	41 (63.1)	16 (72.7)
Leukocytosis, N (%)	8 (12.3)	0 (0)
Leukopenia, N (%)	34 (52.3)	12 (54.5)
Neutropenia, N (%)	28 (43.1)	11 (50)
Lymphopenia, N (%)	39 (60)	11 (50)
Monocytopenia, N (%)	20 (30.8)	9 (40.9)
Thrombocytosis, N (%)	2 (3.1)	1 (4.5)
Thrombocytopenia, N (%)	36 (55.4)	9 (40.9)
Paraproteinemia, N (%)	12 (18.5)	2 (9.1)
Presence of vacuoles, N (%)	27 (62.8)*	6 (50)**
*UBA1* gene mutations		
p.Met41Thr, N (%)	26 (40)	10 (45.5)
p.Met41Leu, N (%)	12 (18.5)	8 (36.4)
p.Met41Val, N (%)	16 (24.6)	3 (13.6)
Other mutations#, N (%)	11 (16.9)	1 (4.5)

*Assessed in 43 patients; **assessed in 12 patients; # Noncanonical, non-Met41 UBA1 mutations included p.Gly40_Lys43del (n = 5), p.Ile894Ser (n = 2), p.Gly477Ala (n = 2), and p.Ser56Phe (n = 2) in patients with recurrent fever episodes. The only noncanonical, non-Met41 mutation observed in patients without fever was p.Ala478Ser.

Acronyms: IQR, interquartile range; N, number (absolute frequency); SD, standard deviation.

Bold values are statistically significant values.

**Figure 1:**
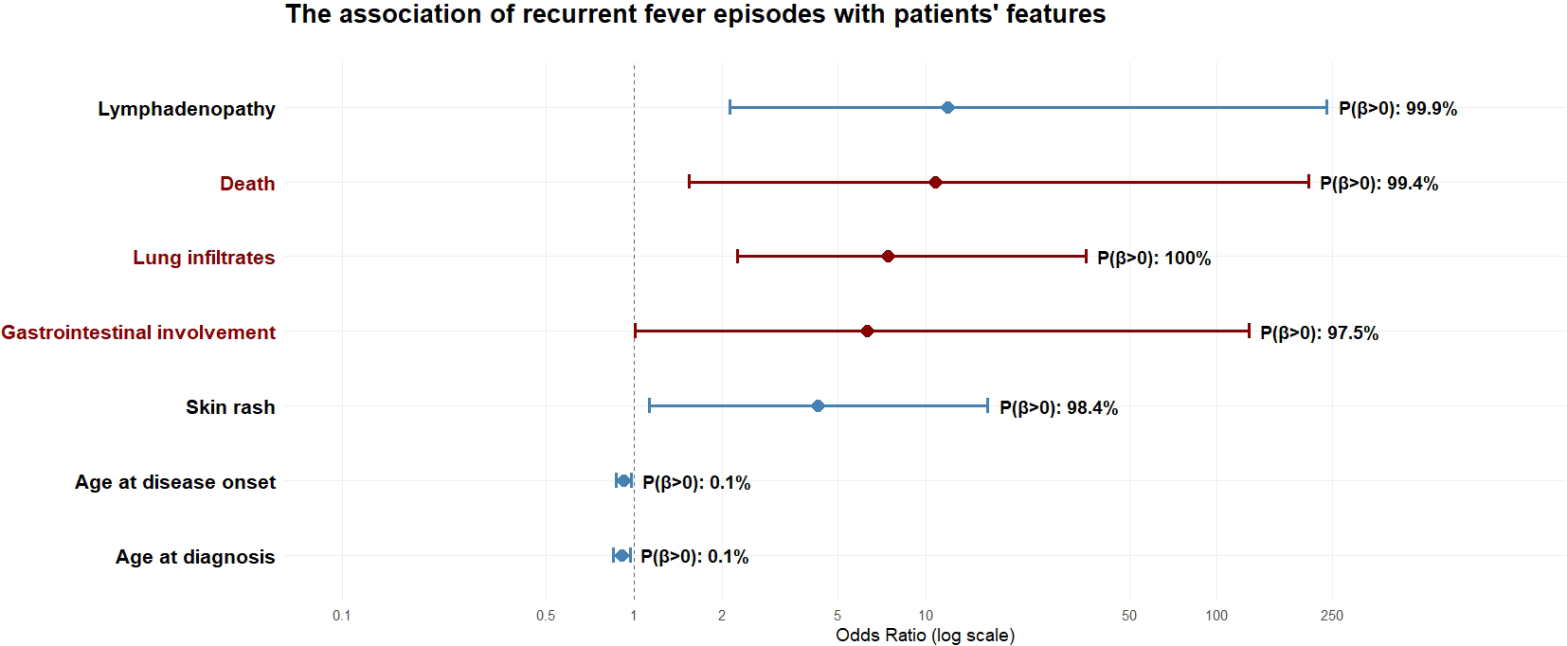
Forest plot summarizing the results of the Bayesian regression analysis evaluating the association between recurrent fever episodes and selected demographic variables, clinical manifestations, and death. Odds ratios are shown on a logarithmic scale with corresponding 95% credible intervals. Variables with estimates affected by perfect separation or very small sample size were not displayed due to limitations of graphical representation. Major organ involvement and death are highlighted in dark red. For each variable, the posterior probability of a positive association with recurrent fever episodes (P(β>0)) is reported on the right.

**Table 2** reports the ORs, 95% CrIs, and the posterior probabilities for the association of fever with demographic, clinical, and therapeutic features. In particular, fever showed a significant association with mortality [P(β): 99.41%], as well as with major inflammatory organ involvement, including cardiac [P(β): 99.99%] and lung [P(β): 99.98%]. At the threshold of significance, gastrointestinal involvement [P(β) = 97.5%] was associated with the presence of fever episodes. Lymphadenopathy, pericardial effusion, uveitis, eyelids edema, abdominal pain accounted for additional clinical manifestations associated with recurrent fever episodes [P(β)>97.5% for each clinical manifestation]. **Figure 1** illustrates the key associations between the occurrence of fever episodes and disease manifestations, with a focus on mortality and major organ inflammation.

**Table 2:** Bayesian regression analysis with the variables listed in the first column as dependent variables and the presence of fever as the independent variable.

Variable	OR (95% CrI)	P(β>0)
Age at disease onset	**0.92 (0.87-0.98)**	**0.14%**
Age at diagnosis	**0.91 (0.85-0.97)**	**0.13%**
Relapsing-remitting course	2.52 (0.88-7.86)	95.57%
Continuous (chronic) course	0.4 (0.1-1.34)	6.88%
Number of attacks/year	1.23 (0.94-2.14)	88.37%
Duration of attacks	0.98 (0.76-1.27)	43.3%
**Death**	**10.81 (1.54-206.4)**	**99.41%**
**Skin rash**	**4.27 (1.13-16.37)**	**98.45%**
Neutrophilic dermatosis	2.7 (0.75-12.59)	93.4%
Macular lesions	0.97 (0.23-5.01)	48.54%
Urticarial papules	1.13 (0.22-7.98)	55.94%
Urticarial like eruptions	3.76 (0.59-88.22)	90.52%
Erythema	6.03 (0.99-148.4)	97.4%
**Eyelids edema**	**2029 (2.02-1.68x10^20^)**	**99.17%**
Other typed of skin rash	107.46 (0-1.40x10^42^)	70.6%
Ocular involvement	1.46 (0.55-4.09)	77.39%
**Uveitis**	**4.94x10^20^ (9.68-2.39x10^80^)**	**99.7%**
Scleritis	0.99 (0.23-4.9)	47.1%
Episcleritis	4.18 (0.51-91.8)	88.6%
Periorbital edema	1.45 (0.38-6.8)	68.2%
Arthritis	1.36 (0.51-3.1)	72.56%
Chondritis	242 (0-2.97x10^60^)	71.28%
**Gastrointestinal involvement**	**6.32 (1.01-128.7)**	**97.5%**
**Abdominal pain**	**e^23.85^ (7.6-1.34x10^42^)**	**99.88%**
Splenomegaly	1.44 (0.46-5.05)	74.12%
Neurological involvement	1.79 (0.48-8.9)	80.19%
Vascular involvement	1.75 (0.66-4.8)	86.98%
**Cardiac involvement**	**6765 (7.68-2.09x10^20^)**	**99.99%**
**Lymphadenopathy**	**11.9 (2.13 - 238)**	**99.88%**
Thoracic pain	3.01 (0.71-21.83)	92.51%
Pleural effusion	4.87 (0.78-123.9)	95.23%
**Pericardial effusion**	**1211 (1.22-4.94x10^18^)**	**98.02%**
**Lung infiltrates**	**7.44 (2.26-35.5)**	**99.98%**
Kidney involvement	1.18 (0.24-8.86)	57.57%
Concomitant haematological disorders	1.02 (0.37-2.68)	51.41%
Myelodysplastic syndrome	0.69 (0.26-1.83)	22.68%
Systemic comorbidities	0.85 (0.31-2.2)	36.5%
Laboratory items		
**Proteinuria**	**903 (1.15-6.9x10^18^)**	**98.01%**
Anemia (all types)	1.06 (0.14-5.34)	52.54%
Macrocytic anemia	0.79 (0.28-2.1)	32.29%
Macrocytosis	0.73 (0.24-2.0)	28.23%
**Leukocytosis**	**1480 (2.55-4.1x10^17^)**	**99.58%**
Leukopenia	1.0 (0.39-2.66)	50.41%
Neutropenia	0.82 (0.32-2.2)	34.22 %
Lymphopenia	1.64 (0.63-4.28)	84.59%
Monocytopenia	0.69 (0.25-1.93)	23.25 %
Thrombocytosis	0.86 (0.08-20.86)	45.09%
Thrombocytopenia	1.96 (0.74-5.37)	91.27%
Paraproteinemia	2.7 (0.63-20.1)	89.83%
Presence of vacuoles	2.1 (0.74-6.63)	91.58%
*UBA1* gene mutations		
M41T	0.82 (0.3-2.31)	34.67%
M41L	0.32 (0.1-1.00)	2.54%
M41V	2.06 (0.55-9.97)	84.95%
**Non M41 mutations**	**2798 (3.38-3.78 x10^18^)**	**99.95%**

Odds ratios (ORs) with 95% credible intervals (CrIs) and posterior probabilities are reported. The posterior probability [P(β)] indicates the probability that fever is associated with the presence of the given feature in this study population. Associations were considered significant when P(β > 0) exceeded 97.5%.

Bold values are statistically significant values.

**Supplementary Table 1** provides detailed posterior probabilities for the association between fever episodes of varying intensity (37.1-37.7 °C, 37.8-40 °C, >40 °C) and the development of different clinical manifestations. Notably, mortality was most likely among patients experiencing fever episodes exceeding 40 °C [P(β): 99.8%]. While fever was associated with cardiac involvement regardless of body temperature, pulmonary [P(β): 99.45% 37.8-40 °C and 99.84% for >40 °C], gastrointestinal [P(β): 99.81% 37.8-40 °C and 98.02% for >40 °C], and cutaneous involvement [P(β): 99.88% 37.8-40 °C and 99.8% for >40 °C] were primarily linked to episodes exceeding 37.7 °C. Fever episodes with temperatures 37.1-37.7 °C and 37.8-40 °C were associated with the development of uveitis [P(β):99.8% and 98.3%, respectively], and pericardial effusion [P(β): 99.5% and 99.9%, respectively]. Proteinuria was more frequently observed in patients experiencing fever episodes with temperatures 37.1–37.7 °C [P(β): 99.78%]. Additionally, the association between fever episodes and age at disease onset was most pronounced in patients with temperatures between 37.8 and 40 °C. **Supplementary Figure 1** graphically depicts how the posterior probability of associations between specific clinical features in VEXAS patients varies with different levels of fever.

While **Table 3** provides information on the frequency of cDMARDs, bDMARDs, and JAK inhibitors used in this cohort, **Table 4** reports the associations between fever and treatment response, categorized as complete response, partial response, or treatment failure. Notably, the presence of fever was not directly associated with either complete response or treatment failure. However, for cDMARDs, JAK inhibitors, and tocilizumab, the occurrence of recurrent fever episodes was associated with a negligible probability of both complete response and treatment failure [P(β) <2.5%], instead favoring a partial response [P(β) >97.5%].

**Table 3:** Frequency of use of conventional disease modifying anti-rheumatic drugs (bDMARDs), biotechnological agents (bDMARDs), and janus Kinases (JAK) inhibitors in patients with and without recurrent fever episodes.

Variables	Patients with fever, N=65	Patients without fever, N=22
cDMARDs	53 (81.5)	9 (40.9)
Azathioprine	7 (10.8)	0 (0)
Colchicine	7 (10.8)	0 (0)
Cyclosporine	5 (7.7)	0 (0)
Hydroxychloroquine	6 (9.2)	0 (0)
Leflunomide	2 (3.1)	1 (4.5)
Mesalazine	2 (3.1)	0 (0)
Methotrexate	21 (32.3)	8 (36.4)
Mycophenolate Mofetil	2 (3.1)	0 (0)
Sulfasalazine	1 (1.5)	0 (0)
JAK inhibitors	29 (44.6)	5 (22.7)
Baricitinib	7 (10.8)	0 (0)
Filgotinib	2 (3.1)	0 (0)
Ruxolitinib	11 (16.9)	1 (4.5)
Tofacitinib	4 (6.2)	4 (18.2)
Upadacitinib	5 (7.7)	0 (0)
bDMARDs	49 (75.4)	11 (50)
Abatacept	1 (1.5)	0 (0)
Adalimumab	3 (4.6)	0 (0)
Anakinra	13 (20)	2 (9.1)
Canakinumab	5 (7.7)	0 (0)
Etanercept	5 (7.7)	2 (9.1)
Infliximab	2 (3.1)	1 (4.5)
Rituximab	2 (3.1)	0 (0)
Sarilumab	1 (1.5)	0 (0)
Tocilizumab	17 (26.2)	6 (27.3)

**Table 4:** Bayesian regression analysis of treatment response (complete response, partial response, failure) according to the presence of recurrent fever.

Variables	Complete response	Partial response	Failure
**cDMARDs**	68.5% (2.1, 0.21-46.5)	**98.99% (7.54, 1.35-53)**	**0.3% (0.09, 0.014-0.49)**
Methotrexate	88.7% (4.85, 0.45-104.6)	90% (3.86, 0.52-35.9)	**0.28% (0.03, 0.009-0.4)**
**JAK inhibitors overall**	**0.4% (0.03, 0.001-0.4)**	**98.3% (14.7, 1.2-296)**	96.7% (4.52x10^12^, 0.66-2.02x10^56^)
JAK2 inhibitors	25.2% (5.87x10^-16^, 3.3x10-^44^-18.7x10^3^)	67.5% (3394.8, 2.16x10^-7^-3.01x10^26^)	16.7% (1.7x10^-18^, 9.1x10^-94^-1.3x10^3^)
Non-JAK2 inhibitors	**8.3x10^-5^% (2.1x10^-9^, 3.8x10^-25^-0.021)**	**99.3% (50.4, 2-3463)**	**96.84% (1.14x10^15^, 0.7-1.2x10^61^)**
Ruxolitinib	20.2% (4.3x10^-12^, 4.9x10^-52^ – 12.9x10^3^)	68.8% (12210, 5.14x10^-7^- 3.5x10^28^)	20.2% (4.3x10^-12^, 4.9x10^-52^ – 12.9x10^3^)
**Anti-IL-1 Biotechnological agents**	4.9% (0.005, 1.9x10^-9^-1.99)	89.4% (672, 0.19-4.76x10^11^)	89.5% (1312, 0.16-1.04x10^37^)
Anakinra	3.4% (0.004, 1.9x10^-7^-1.35)	89.6% (4023, 0.18-6.48x10^16^)	89.7% (336, 0.2-5.9x10^10^)
**Tocilizumab**	**2.33% (0.09, 0.006-0.96)**	**97.63% (11.6, 1.04-161)**	NA*

*No failures were observed in patients receiving Tocilizumab.

Reported are posterior probabilities, odds ratios, and 95% credible intervals. While some credible intervals are extremely wide due to sparse data and limited sample size in certain subgroups, the posterior probabilities provide robust probabilistic evidence of differential response patterns. These findings should therefore be interpreted with due caution but also with the recognition that they may reflect meaningful biological and therapeutic signals.

Bold values are statistically significant values.

**Supplementary Table 2** reports the P(β) for the likelihood that each treatment was associated with complete response, partial response, or treatment failure, stratified by the maximum body temperature recorded during febrile episodes (≤37.7 °C, 37.8–40.0 °C, >40.0 °C). Notably, for temperatures exceeding 40 °C, the use of anti-IL-1 agents was associated with a low probability of complete response [P(β): 0.6%] and, conversely, with a high probability of treatment failure [P(β): 99.3%]. JAK inhibitors overall were likewise associated with a low probability of complete response [P(β): 1.7%]. In contrast, JAK2-selective inhibitors appeared associated with complete response [P(β): 99.99%]. At the opposite end of the spectrum, when maximum body temperature ranged between 37.1 and 37.7 °C, both tocilizumab and JAK inhibitors were associated with a low probability of complete response [P(β): 1% and P(β): 4.6%, respectively], while the probability of achieving a partial response was high [P(β): 98.8% and P(β): 95.3%, respectively].

## Discussion

VEXAS syndrome represents a prototypical systemic inflammatory disorder, in which febrile episodes are frequently observed, displaying either a continuous or a relapsing-remitting pattern. Notably, beyond infectious triggers, fever has been reported in at least two-thirds of VEXAS patients, making it one of the most commonly encountered clinical manifestations (5, 16, 20). In this context, the present study aimed to investigate the association between febrile episodes and specific clinical and laboratory features of the disease, as well as their association with demographic characteristics, genetic mutations, and treatment response.

Notably, in the present study, the occurrence of febrile episodes in VEXAS syndrome was associated with increased mortality, particularly when body temperature exceeded 40 °C. Fever was also linked to major organ inflammatory involvement, including cardiac manifestations and pulmonary infiltrates, especially among patients with temperatures above 37.7 °C. Thus, the presence of fever in VEXAS patients appears to delineate a distinct clinical pattern characterized by a higher likelihood of severe organ involvement and, ultimately, an increased risk of death.

Ocular involvement, particularly uveitis and eyelid edema, along with gastrointestinal inflammatory manifestations, all previously reported to be associated with mortality (5, 21), represent additional clinical features linked to febrile episodes in VEXAS syndrome. In particular, mortality was observed more frequently among patients with inflammatory ocular involvement (21), while gastrointestinal disease was recognized early on as being associated with mortality (5).

Skin manifestations, lymphadenopathy, leukocytosis, and proteinuria were also associated with fever in VEXAS syndrome and may represent clinical features indicative of greater disease severity.

Age at disease onset, and consequently age at diagnosis, was found to be inversely associated with fever, as younger patients appeared more likely to experience febrile episodes, particularly with body temperatures ranging between 37.7 and 40 °C. The observed association between febrile episodes and risk of death may therefore be of considerable clinical relevance, since, according to the present study, it is precisely younger patients who were more frequently characterized by febrile manifestations.

Regarding mutations, the p.Met41Leu tended to be significantly associated with the lack of recurrent fever episodes, resembling what previously reported by other studies (5). Noteworthy, in the present study non-canonical-Met41 mutations were found significantly associated with fever. Current evidence indicates that many atypical, non-Met41 *UBA1* mutations cause less potent and differently distributed UBA1 dysfunction than Met41 variants, resulting in milder or atypical autoinflammation due to disruption of a domain essential for ubiquitin activation, leading to a consequent reduction of UBA1b enzymatic activity. Fever is generally considered less frequent in these cases (22, 23). Nevertheless, in the present study, fever was significantly associated with atypical *UBA1* mutations, potentially reflecting a mutation-specific propyretic cytokine dysregulation. In this regard, some noncanonical *UBA1* variants appear to act via pathogenetic mechanisms distinct from canonical mutations, which typically block production of the cytoplasmic UBA1b isoform (2). In particular, these variants might alter messenger RNA splicing, generating multiple aberrant transcripts and a modified, yet not fully truncated, protein. Thus, non-Met41 mutations can converge on the UBA1 dysfunction axis, producing a phenotype consistent with VEXAS syndrome, including recurrent fever (24). Whether and to what extent the different pathogenetic mechanisms influence the febrile component still needs to be clarified in larger future studies. Indeed, the limited number of patients included in the present cohort prevented a reliable assessment of the association between non-Met41 mutations and the degree of body temperature elevation.

With the limitation of the relatively small sample size, this study also investigated the potential association between febrile episodes and treatment response. The results suggested that fever is consistently associated with only a partial response to cDMARDs, regardless of the degree of temperature elevation. A similar pattern was observed among patients treated with small molecules: when stratifying patients according to peak temperature, the presence of fever episodes between 37.8 and 40 °C was associated with therapeutic failure. Given the established evidence that JAK2 inhibitors achieve superior efficacy in VEXAS patients compared with other JAK inhibitors (10, 11), a distinction was introduced in the present analysis. This revealed that fever was associated with only a partial response to non-JAK2 inhibitors, particularly among patients with body temperature up to 37.7 °C, whereas the association with complete response was weak. Conversely, for JAK2 inhibitors no statistically significant association could be demonstrated between fever and treatment response, although body temperature exceeding 40 °C was significantly associated with a complete response. Regarding anti-IL-1 biologic agents, particularly anakinra, fever with body temperature exceeding 40 °C was associated with therapeutic failure. In contrast, treatment with the anti-IL-6 agent tocilizumab was linked to only a partial response in the presence of febrile episodes, especially in the subgroup of patients with body temperature not exceeding 37.7 °C.

This study provides clinically relevant insights, yet several limitations should be acknowledged. Firstly, the wide 95% credible intervals observed for several variables in Bayesian models reflect the limited sample size, low event frequency, and occasional imbalance between groups. While this generates uncertainty regarding the precise magnitude of effect, the posterior probabilities still provide meaningful insight into potential associations between fever and clinical, laboratory, and therapeutic features. Therefore, it is important to note that these results are exploratory and intended to generate hypotheses rather than provide definitive conclusions. Indeed, the study’s strength lies in its comprehensive evaluation of a rare condition, integrating both clinical and laboratory data within a Bayesian framework, which allows for probabilistic interpretation even in the context of sparse data. Future studies with larger cohorts and more frequent events are required to obtain more precise effect estimates and to confirm these preliminary findings.

In the present study, patients with temperature elevations below 38.0 °C were also included. This methodological choice was made to ensure a more comprehensive evaluation of disease activity, acknowledging that even mild increases in body temperature may represent a clinically relevant expression of systemic inflammation. Accordingly, to better evaluate the associations between body temperature elevations and clinical manifestations or treatment responses, a more detailed analysis was performed to determine which degrees of temperature increase were associated with specific clinical features or therapeutic outcomes. Additional concerns relate to the retrospective design and the multicenter, registry-based enrollment, which may introduce heterogeneity in clinical data collection, variability in data completeness, and a potential risk of selection bias inherent to observational registries. Also, although follow-up duration did not significantly differ between patients with and without fever episodes, we acknowledge that the retrospective design does not allow full control for time-dependent biases in the assessment of outcomes and treatment response. Finally, the limited number of patients receiving azacitidine precluded the evaluation of treatment response in relation to febrile episodes. Despite these limitations, and although these findings are exploratory and not intended to directly change therapeutic algorithms, they may support clinical risk stratification and help identify patient subgroups deserving closer monitoring and tailored therapeutic strategies. Beyond confirming fever as a frequent manifestation potentially related to more severe disease, our findings provide a temperature-graded clinical stratification showing differential associations with major organ involvement, mortality risk, and patterns of treatment response.

In conclusion, febrile episodes, a relatively frequent clinical manifestation in patients with VEXAS syndrome, are associated with more severe pattern of organ involvement, including inflammatory manifestations of the pulmonary, gastrointestinal, and cardiac systems. Accordingly, the occurrence of fever episodes is associated to death in VEXAS syndrome. Furthermore, the presence of febrile episodes could correlate with differential therapeutic responsiveness, thereby potentially serving as a valuable marker to guide the most appropriate treatment strategies in VEXAS patients.

## Data Availability

The raw data supporting the conclusions of this article will be made available by the authors, without undue reservation.

## Supplementary material

Supplementary Figure 1 Heatmap of the posterior probability of association between fever intensity and clinical features. The heatmap displays the association between different ranges of maximum temperature during flares (x-axis) and specific clinical variables (y-axis). Colours represent the posterior probability percentage, with higher probabilities shown in darker red.
