# Supplementary figures and images for: Recurrent fever and association with severe organ involvement, mortality and treatment outcomes in VEXAS syndrome: data from the AIDA Network
Source: Front Immunol. 2026 Mar 13;17:1753412. doi: 10.3389/fimmu.2026.1753412 (PMC13021879; doi:10.3389/fimmu.2026.1753412)

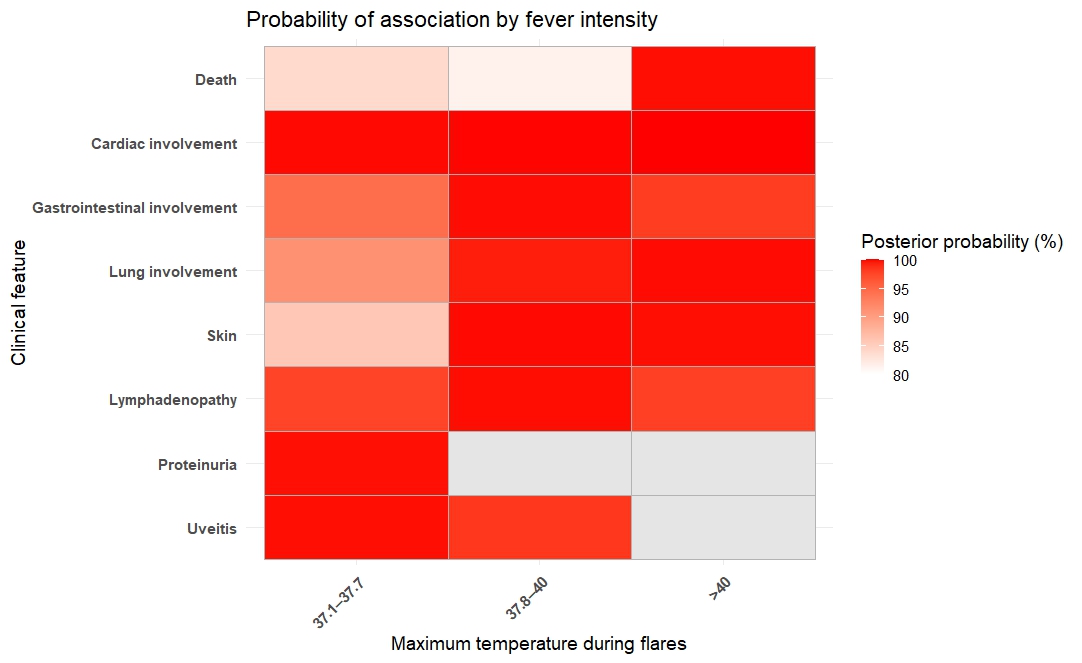

Supplement: Supplementary Figure 1 — Heatmap of the posterior probability of association between fever intensity and clinical features. The heatmap displays the association between different ranges of maximum temperature during flares (x-axis) and specific clinical variables (y-axis). Colours represent the posterior probability percentage, with higher probabilities shown in darker red. [file Image1.jpeg]
