# Supplementary material for: Recurrent fever and association with severe organ involvement, mortality and treatment outcomes in VEXAS syndrome: data from the AIDA Network
Source: Front Immunol. 2026 Mar 13;17:1753412. doi: 10.3389/fimmu.2026.1753412 (PMC13021879; doi:10.3389/fimmu.2026.1753412)
Supplement: Supplementary file 2 [file Table1.docx]

**Supplementary Table 1.** Posterior probabilities of each clinical manifestation significantly associated with the occurrence of fever, stratified by fever intensity (37.1-37.7 °C, 37.8-40.0 °C, and >40.0 °C). Posterior probabilities >97.5% or <2.5% are highlighted in bold and underlined to denote statistical significance.

|  | **37.1- 37.7°C** | **TC 37.8°C-40.0°C** | **Beyond 40.0°C** |
| --- | --- | --- | --- |
| **Age at disease onset** | 3% | **0.003%** | 22.1% |
| **Age at diagnosis** | 3.2% | **0.003%** | 13.3% |
| **Skin manifestations** | 85.72% | **99.88%** | **99.8%** |
| **Death** | 83.9% | 81.5% | **99.8%** |
| **Cardiac involvement** | **99.88%** | **99.95%** | **100%** |
| **Pericardial effusion** | **99.5%** | **99.9%** | 44.4% |
| **Gastrointestinal involvement** | 94.47% | **99.81%** | **98.02%** |
| **Abdominal pain** | 44.5% | **99.9%** | 36.3% |
| **Lung infiltrates** | 91.23% | **99.45%** | **99.84%** |
| **Lymphadenopathy** | **97.59%** | **99.83%** | **97.84%** |
| **P****roteinuria** | **99.78%** | 56.38% | 17.27% |
| **Uveitis** | **99.8%** | **98.3%** | 34.4% |
| **Eyelids edema** | 44.89% | **97.68** | **98.46** |
| **Non-M41 mutations** | 47.9% | 36.4% | 43.8% |

**Supplementary Table 2**. Posterior probabilities for the association between different degrees of fever (37.1-37.7 °C, 37.8-40.0 °C, >40.0 °C) and treatment outcomes (complete response, partial response, or failure). Posterior probabilities >97.5% or <2.5% are highlighted in bold and underlined as statistically significant. *Abbreviations: °C, Celsius degrees; cDMARDs, conventional disease-modifying antirheumatic drugs; JAK, Janus kinase; IL, interleukin; MTX, methotrexate.*

|  |  | **37.1- 37.7°C** | **37.8-40.0°C** | **Beyond 40.0°C** |
| --- | --- | --- | --- | --- |
| **cDMARDs** | **Complete response** | 46.4% | 52.2% | 8.9% |
|  | **Partial response** | **99.1%** | **97.7%** | **99.3%** |
|  | **Failure** | **0.9%** | **2.2%** | **0.6%** |
| **MTX** | **Complete response** | 70.3% | 71.4% | 22% |
|  | **Partial response** | 95.8% | 80.2% | 94.9% |
|  | **Failure** | **0.2%** | 9.4% | 3.1% |
| **JAK inhibitors overall** | **Complete response** | 4.6% | **0.4%** | **1.7%** |
|  | **Partial response** | 95.3% | 95.9% | 49.1% |
|  | **Failure** | 38.3% | **99.5%** | 55.5% |
| **JAK2 inhibitors** | **Complete response** | 91.5% | 91.4% | **99.99%** |
|  | **Partial response** | 7.4% | 7.7% | **0.001%** |
|  | **Failure** | 92.3% | 90.7% | 64.2% |
| **Non-JAK2 inhibitors** | **Complete response** | **0.008%** | **0.003%** | 10.2% |
|  | **Partial response** | **99.8%** | 96.5% | **98.2%** |
|  | **Failure** | 46.2% | **99.8%** | 58.8% |
| **Ruxolitinib** | **Complete response** | NA* | 75.1% | NA* |
|  | **Partial response** | NA* | 22.35% | NA* |
|  | **Failure** | NA* | 72.6% | NA* |
| **Anti-IL-1 agents** | **Complete response** | 4.1% | 9% | **0.6%** |
|  | **Partial response** | 93.4% | 64.5% | 31.7% |
|  | **Failure** | 20.8% | 76.1% | **99.3%** |
| **Anakinra** | **Complete response** | 7.2% | 7.3% | **0.3%** |
|  | **Partial response** | 92.2% | 64.1% | 34.5% |
|  | **Failure** | 31% | 80.9% | **99.3%** |
| **Tocilizumab** | **Complete response** | **1%** | 6.4% | 3.2% |
|  | **Partial response** | **98.8%** | 93.2% | 96.9% |
|  | **Failure** | NA# | NA# | NA# |

**NA*** Only patients experiencing fever episodes of 37.8–40.0 °C were treated with ruxolitinib.
**NA#** No patients treated with tocilizumab experienced treatment failure.
